# Mesoporous Silica Materials as Drug Delivery: “The Nightmare” of Bacterial Infection

**DOI:** 10.3390/pharmaceutics10040279

**Published:** 2018-12-15

**Authors:** Marina Martínez-Carmona, Yurii K. Gun’ko, María Vallet-Regí

**Affiliations:** 1 School of Chemistry and CRANN, Trinity College, The University of Dublin, Dublin 2, Ireland; igounko@tcd.ie; 2 Department Chemistry in Pharmaceutical Sciences, School of Pharmacy, Universidad Complutense de Madrid, Instituto de Investigación Sanitaria Hospital 12 de Octubre i+12, 28040 Madrid, Spain; 3 Centro de Investigación Biomédica en Red de Bioingeniería, Biomateriales y Nanomedicina (CIBER-BBN), Avenida Monforte de Lemos, 3-5, 28029 Madrid, Spain

**Keywords:** mesoporous silica, drug delivery, bacterial infection, bacterial biofilm, biofilm, antibiotic resistance, targeting bacteria, targeting biofilm, multifunctional nanoparticles

## Abstract

Mesoporous silica materials (MSM) have a great surface area and a high pore volume, meaning that they consequently have a large loading capacity, and have been demonstrated to be unique candidates for the treatment of different pathologies, including bacterial infection. In this text, we review the multiple ways of action in which MSM can be used to fight bacterial infection, including early detection, drug release, targeting bacteria or biofilm, antifouling surfaces, and adjuvant capacity. This review focus mainly on those that act as a drug delivery system, and therefore that have an essential characteristic, which is their great loading capacity. Since MSM have advantages in all stages of combatting bacterial infection; its prevention, detection and finally in its treatment, we can venture to talk about them as the “nightmare of bacteria”.

## 1. Introduction

Despite living in a society increasingly concerned about hygiene and asepsis, in reality, we live in a world populated by microorganisms that can be found in the most unsuspected places, such as the door handle, the mobile phone, or on the kitchen sponge [1,2,3,4]. Although some of them do not cause any serious harm or can even be beneficial [5,6], others can be highly damaging, compromising our health, and even causing death. Since the mid-20th century, society has become aware of this, and has begun developing numerous antibiotics that can efficiently fight bacteria and greatly reduce mortality from infections. However, in recent years, this trend has begun to change, mainly due to an unjustified and continued use of antibiotics that is forcing bacteria to develop resistance against common antibacterial drugs [7,8]. In 2016, the European Food Safety Authority (EFSA) and the European Centre for Disease Prevention and Control (ECDC) conducted a study in 28 European member states. This study demonstrates an increased resistance to certain antibiotics in *Salmonella*, *Campylobacter*, *Escherichia coli*, and *Staphylococcus aureus* bacteria [9]. 

In addition to this acquired resistance, bacteria have an innate self-defense mechanism called biofilm formation [10]. Biofilms are defined as communities of microorganisms that grow adhered to a surface, and that are embedded in a protective self-produced extracellular matrix. This gives them certain and special characteristics, making them different from bacteria in a planktonic state (that is, as individual cells floating in solution), and able to play the important role of providing an increased resistance to antibiotics [11]. 

Combined together, these two phenomena; having an acquired resistance to antibiotics and making biofilm formations, reduce bacterial sensitivity to antibiotics and hinder the effectiveness of medication. As a consequence, in order for the antibiotic to be effective, it is necessary to increase the dose and frequency of the treatments, favoring again the appearance of resistant bacteria; a vicious circle that seems to have no end. This is not to mention the side effects, such as hypersensitivity, the effects of commensal flora, or the appearance of opportunistic pathogens in the human body that can be caused by an abusive and prolonged consumption of antibiotics [12]. As a result, it has become necessary to explore new and more effective paths to the early detection and treatment of bacterial infections. Numerous efforts are being made to find alternatives such as the use of predatory bacteria [13], bacteriophages [14], bacteriocins [15], probiotics and prebiotics [16,17], etc. However, until now, none of them has shown efficacy that is comparable to that of antibiotics.

Of course, another solution is the design of new antibiotics to which the bacteria are not yet resistant. In fact, according to a recent report by PEW, currently there are 45 new antibiotics in different phases of the clinical study [18]. The problem is that without a change of behavior regarding the use of antibiotics, after a short time, these drugs will stop being effective, and the vicious circle would return to its beginning. Therefore, the solution is not to design new drugs continuously, but to increase the durability and effectiveness of those that are known. This can be accomplished by the use of nanomaterials (NMs), which in recent years, have proven to be a great alternative in the treatment of infection and other diseases [19,20].

Nanomaterials, with at least one of their dimensions in the order of nanometers, have a characteristic size that is halfway between the molecules and bulk materials, which allows them to interact uniquely with bacteria. In many cases, when NMs are in contact with the bacteria, they can disrupt the bacterial cell wall with no need for internalization, resulting in its toxicity, due to a simple matter of size. However, the way in which each type of NMs destroys or affects bacteria is extremely dependent on the NM’s composition, as well as the strain of the bacteria to be treated. In general, for metal NMs such as gold, or silver, their toxicities lie mainly in their ability to release metal ions into the environment, generate reactive oxygen species (ROS), or produce some photothermal effects [21]. Similarly, the use of some TiO_2_ or ZnO quantum dots for antibacterial treatment are due to their ability to produce ROS, especially after UV light irradiation [22]. Cationic peptides are also used with antimicrobial purposes taking advantage of its hydrophobicity, and especially its cationic nature that is able to physically damage the bacterial membrane through electrostatic interactions [23]. Recently, more and more importance is being given to the use of different drug delivery nanomaterials, and although they may not have a clear antibiotic effect by themselves, they do allow for the loading and release of antibiotics when needed, enhancing their therapeutic capacity [24,25]. Liposomes [26,27], polymeric nanoparticles [28], and mesoporous silica materials (MSM) are some of the most common nanocarriers that used for this purpose. Despite their great biocompatibility, liposomes present poor stability and low loading capacity. On the other hand, polymeric nanoparticles usually present a fast release of the cargo, due to the degradation of the polymer shell, hindering their application for prolonged treatments [29]. Both issues can be overcome by the use of MSM, which are robust materials that remain stable for long periods, and that have a great surface area and high pore volume, meaning that they have a large loading capacity [30]. In addition, MSM allow for the achievement of high tunability at different levels; see below.

### 1.1. Tunability of the Porous Structure

**Figure 1:**
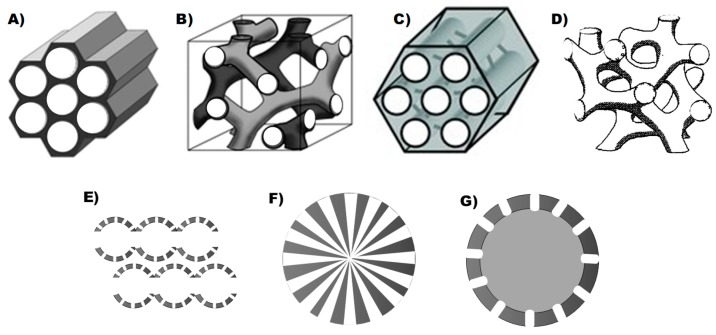
The most common porous silica structures for drug delivery: (**A**) hexagonal MCM-41; (**B**) cubic MCM-48 (adapted with permission from [33], copyright Wiley 2006); (**C**) hexagonal with microporous connections SBA-15 (adapted with permission from [34], copyright Wiley 2012); (**D**) disorder LMU-1 (adapted with permission from [35], copyright American Chemical Society 2008); (**E**) mesocellular foam IBN-3 [36,37]; (**F**) dendritic pores [38]; (**G**) mesoporous hollow particles [39].

By varying the synthesis conditions and the nature of the reagents, it is possible to modify the structure, composition, and size of the pores, adapting them to the requirements [31,32]. This is of great importance when designing a controlled release system. Thus, depending on the drug to be loaded, it may be necessary to increase or decrease the size of the pore to avoid steric impediments. By modifying the nature of the functional groups of the silica network, it is possible to control the drug/matrix interactions. In addition, the type of porous structure affects the process of adsorption/desorption of the drug. The most common porous silica structures for drug delivery are shown in Figure 1, with MCM-41 being the most frequent for the treatment of bacterial infection, according to the literature. 

All of these factors having a great influence on the loading capacity and the release kinetics of the drug. Thanks to this versatility, looking for the right combination of the different parameters (size, interaction type, and porous structure) it is possible to use MSM to load and release any antibiotic, drug, or other type of molecules.

### 1.2. Tunability of the Shape of the Nanodevice

MSM can be modified from a bulk material into a well-controlled size of nano- or micrometer particles. Their shape can also be changed into the form of rods [40], oblates [41], or their most common, spherical shapes, amongst many other forms. Several studies have been performed on how the shape and the size of MSM affects its interaction with cells [42,43]. However, there are only a few studies on how these parameters affect bacteria. Rezwan et al. studied the effect on the viability of *B. subtilis*, *B. megaterium*, and *E. coli* when exposing them to three different sizes of silica particles (15, 50 and 500 nm), observing no significant reduction of colony forming units (CFU) compared to the control [44]. Recently, T. Cohen-Bouhacina et al. suggested that only counting CFU might be insufficient to probe any toxicity as it could be affected by splitting effects [45]. After carrying out atomic force microscopy (AFM) experiments on *E. coli* bacteria with silica nanoparticles between 4 and 100 nm, they observed a critical particle diameter in the range 50-80 nm. Thus, while particles below this size produced partial collapse or lysis of the cell membrane, the larger particles did not show any effect on the bacterial morphology. Regarding how the size and shape of silica particles affect biofilms, M. H. Schoenfisch reported that for nitric oxide-releasing silica particles and rods, smaller sizes and higher aspect ratios were more effective in biofilm eradication [46]. Although a deeper study is needed, it seems that both particle size and shape affect bacteria and biofilms, and are therefore important factors to consider when designing a nanocarrier. 

### 1.3. Tunability of the Nature of the Surface

Silica materials have a high number of silanol groups on their surface, which allows us to easily modify the physicochemical nature of the surface through the process of functionalization [47,48], endowing them with great versatility, since it is possible: (i) to include dyes in the structure to detect and follow the evolution of the infection [49,50,51,52]; (ii) to incorporate positive functional groups that produce disruption of the bacteria’s outer membrane [45]; (iii) to produce stimuli-responsive devices [53,54], (iv) to bind molecules in order to obtain antiadherent surfaces that reduce bacterial adhesion, and that therefore create biofilm formation [55]; (v) to tune the surface with targeting molecules that enhance bacteria or biofilm penetration [56].

In addition to all these properties, it has been demonstrated that MSM act as adjuvants, activating the immune response, and therefore, contributing to increasing the effectiveness of the vaccine and its immunological power [57,58].

**Figure 2:**
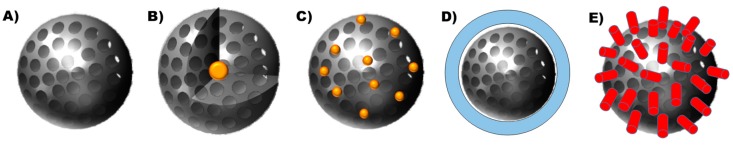
The most common types of mesoporous silica materials (MSM) in the form of nanoparticles (MSNPs) that are used as drug delivery systems: (**A**) bare MSNPs; (**B**) (metal or other material NP core) @ (mesoporous silica shell); (**C**) metal NPs/MSNPs; (**D**) MSNPs coated with a polymer shell and (**E**) molecule functionalized-MSNPs.

Despite all the advantages that MSM present as drug delivery systems (DDS) for the treatment of different pathologies [53,59,60], there is no perfect material that can be used alone for MSM. Increasingly, the scientific community devotes more time to combining different materials whose properties can be combined to achieve more polyvalent devices. In this sense, the combination of mesoporous silica materials with other elements, such as gold or silver nanoparticles, quantum dots, and different polymers or molecules, can produce an antimicrobial synergistic effect. Figure 2 shows the most common models of MSM in the form of nanoparticles (MSNPs) for drug delivery [61]. It is important to highlight that the same models can be presented with other shapes, but the sphere has been chosen for the example, as it is the most commonly used. 

Since MSM have advantages in all stages of combatting bacterial infection, starting with its prevention, through its detection and finally in its treatment, we could venture to talk about them as the “nightmare of bacteria”. However, among all the properties that have been previously discussed, their great loading capacity is one of the main reasons for the growing popularity of MSM, since they were first proposed as DDS [62]. Thus, in this review, we overview the use of MSM acting as DDS in the field of bacterial infection treatment, summarizing their latest advances in combating planktonic bacteria, preventing biofilm formation and destroying the formed biofilm. 

## 2. Effect on Planktonic Bacteria 

**Figure 3:**
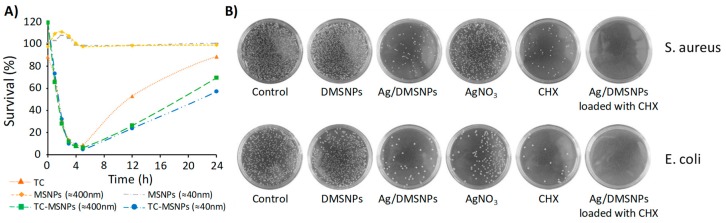
(**A**) Survival (%) of *E. coli* treated with a concentration: 1.0 µg/mL of tetracycline (TC) or its equivalent in loaded MSNPs (adapted with permission from [67], copyright MDPI 2015). (**B**) Photographs of Luria-Bertani (LB)-agar plates coated with *S. aureus* (up) and *E. coli* (below) when supplemented with Ag/dendritic mesoporous silica nanoparticles (DMSNPs) loaded with CHX, CHX, AgNO_3_, Ag/DMSNPs, and DMSNPs, respectively (adapted with permission from [68], copyright Dovepress 2017).

As stated previously, the increasingly common resistance acquired from bacteria leads to higher doses and frequencies of antibiotics with the consequent side effects. The use of DDS emerges as a solution to protect the antibiotics, and to concentrate its action into the target bacteria, therefore improving its effectiveness. According to this, there are a number of different research groups who have chosen to study the increase in the effectiveness of different antibiotics after being loaded onto MSM. Since the number of published works on the loading and release of antibiotics on MSM [63,64,65,66] is very extensive, we are only going to focus on those that include experiments with bacteria. A. J. Di Pasqua et al. conducted an experiment in which they synthesized mesoporous silica nanoparticles (MSNPs) with two different sizes, around 40 and 400 nm, and loaded them with tetracycline (TC), achieving a cargo of 18.7 and 17.7% (*w*/*w*), respectively [67]. After that, they compared the antibacterial capacity of both types of particles with the free drug against *E. coli*. The results showed that during the first few hours there were no differences in bacterial inhibition. However, after four hours, both types of particles proved to have a better antibacterial effect (Figure 3A). According to the authors, this may be due to the fact that by being inside the pores, the drug remains stable for a longer time.

Polymyxin B (PolyB) is a potent antibiotic against resistant Gram-negative bacteria. However, its use is not as widespread as one might expect, since it also presents some toxicity in mammalian cells. A. Arpanaei et al. studied the biocompatibility and antibacterial efficacy of three different types of PolyB loaded MCM-41 MSNPs (bare, aminated, and carboxylated). The results demonstrated that loading the PolyB onto the nanoparticles did not improve the antibacterial capacity, but instead enhanced the biocompatibility of the free drug, especially for MSNPs that are incubated with HEK293 cells [69]. MSNPs have also been used to increase the effectiveness of some essential oils that demonstrate an antibacterial effect, according to reports by E. Andronescu et al. [70].

MSM have an advantage in that we are able to obtain nanoparticles with different sizes and pore diameters through modifying the nature of the surfactant and synthesis conditions [71], meaning that they are able to load not only small molecules, but also larger proteins. C. Yu et al. took advantage of this possibility and synthesized dendritic mesoporous silica nanoparticles (DMSNPs) with different pore sizes, ranging from 2.7 to 22.4 nm [72]. These particles were loaded with lysozyme (lys), a natural protein that is able to cleavage the glycosidic bonds that are present in the cell wall of Gram-positive bacteria. As expected, the DMSNPs with the bigger pore diameter loaded higher amounts of lys (244.5 mg/g), compared with those that had a pore diameter of 2.7 nm, which loaded less amounts (37.6 mg/g). Due to the small size of the pores, the lys is only retained on its surface, and it relies on a kinetic release with a large initial burst. On the other hand, the larger pore samples showed a sustained kinetic effect over time. Finally, the in vitro antibiotic activity of lys-loaded MSNPs was compared with free lys against *E. coli* bacteria. The free lys presented the highest Minimum Inhibitory Concentration (MIC), which was determined to be 2500 mg/mL, while the DMSNPs with bigger pores, presented the lowest MIC (500 µg/mL). In the same way, the NPs with a pore diameter of 22.4 nm proved to be those that produced greater long-term bacterial inhibition and that generated stronger damage in the bacterial wall, according to the Scanning electron microscopy (SEM) images. N. Sriranganathan et al. investigated the antibacterial capacity of gentamicin loaded biodegradable silica xerogel against mice infected with *Salmonella enterica* [73]. With the silica xerogel, there was a clear reduction in infection, both in the spleen and the liver, while no significant reduction was observed with the free drug.

It has been demonstrated that loading antibiotics onto MSNPs produces certain improvements in the effectiveness of the drug. However, the chemical nature of MSM allows the surface to be modified according to its needs, providing these systems with greater versatility. Hence, by combining drug delivery systems, based on MSM with other antimicrobial agents, we can achieve an added value in bacterial eradication. It is well-known that some metals such as copper, silver, nickel, zinc, and others have quite strong antibacterial properties [74,75]. Based on this information, several groups have focused their research on the combination of some of these metals with silica nanoparticles. For example, in 2009 J. Zink et al. reported their findings on the antibacterial effect of silver nanocrystals, encapsulated in MSNPs [76]. According to their results, the silica coating reduced the hydrophobicity of the silver nanocrystals, decreasing their aggregation without compromising the oxidation of the silver crystals that were slowly released in the medium. To study the antimicrobial efficacy of the NPs, two different experiments were performed against two types of bacteria; *Bacillus anthracis*, and *E. coli*, (as Gram-positive and Gram-negative models, respectively). In the first one, three different concentrations of nanoparticles (20, 50, and 100 μg/mL) were added to the agar media, prior to its solidification. The suspension of bacteria was then spread onto the agar plates and incubated overnight. It was observed that the presence of the Ag-coated NPs in the agar prevented both types of bacteria from forming, and this was especially efficient for the *B. anthracis*, even with the smallest concentration. A second experiment studied the effect of the Ag-coated NPs on the bacterial growth kinetics in liquid media. The results showed that the nanoparticles did not produce any significant variation in the growth of *E. coli*; however, its presence slowed *B. anthracis* growth for the concentration of 50 μg/mL, and totally inhibited it at 100 μg/mL. In order to enhance the bacteria-nanoparticle interaction, the surface of the silica was functionalized with different polyelectrolytes, and it was observed that cationic NPs were more effective in slowing the growth of *E. coli*, while surface functionalization had almost no effect on *B. anthracis.* In 2017, Y. Zhou et al. went a step further and studied the synergistic bactericidal effect of loaded chlorhexidine, silver-decorated MSNPs against *S. aureus* and *E. coli* [68]. As can be observed in Figure 3B, the combined treatment was more effective than AgNO_3_ or CHX separately against the *E. coli* and *S. aureus* bacteria. Finally, the biocompatiblity of the system was studied, showing cell mortality that was comparable to that of the controls, while the same concentrations of free CHX or silver ions were clearly toxic. J. Santamaría et al. performed a similar study, but loaded pereacetic acid into the mesopores of SBA-15, containing Ag NPs [77]. 

Other metals whose antibacterial capacities have been studied in combination with silica are copper and nickel. M. Kooti et al studied the following complexes: Mesoporous silica copper-supported nanoparticles (MSNPs-SB-Cu), nickel (MSNPs-SB-Ni), and Schiff base (SB) [78]. After incubating four types of bacteria *S. aureus*, *B. subtilis*, *E. coli*, and *P. aeruginosa* into the presence of the particles, they observed that MSNPs-SB-Cu presented a bacteriostatic effect against *E. coli* and *S. aureus*, and MSNPs-SB-Ni was bacteriostatic against *E. coli*, but bactericidal against *S. aureus.* The authors also loaded the nanoparticles with gentamicin and a performed disc diffusion assay. While non-loaded nanoparticles had almost no effect in any type of bacteria, the gentamicin loaded ones produced a similar halo of inhibition in all cases. A. Meghea et al. combined silica–titanium sieves (Si-Ti-Sv) with izohidrafural (Izo, a new antibacterial agent), and compared their antibacterial performance against different Gram-positive and Gram-negative strains isolated from urinary tract infections [79]. The results obtained showed, once again, that there is no universal treatment. Although in some cases, the combination of Izo-Si-Ti-Sv was the best option (*Klebsiella pneumoniae* and *Proteus mirabilis*) against Gram-positive cocci. The sieves without any load gave better results, and for most *Escherichia coli* strains, the directly administered antibiotic exhibited the highest power. Xiao et al. designed a multifunctional nanodevice that combined: (i) MSNPs to load an antibiotic; (ii) carbon dots (C-dots) to be visualized, and (iii) Rose Bengal (RB), a photosynthesizer that generates ROS. Although initially designed for antitumor purposes, they proved that it was also effective as an antimicrobial agent, especially after loading ampicillin into its pores. The results showed that the combined effect of the antibiotic with the ROS was clearly more effective than any of them separately, especially for a concentration of 100 μg/mL, at which the population of *E. coli* was completely eradicated [80]. All the systems are summarized and referenced in Table 1.

### 2.1. Targeting Bacteria

**Figure 4:**
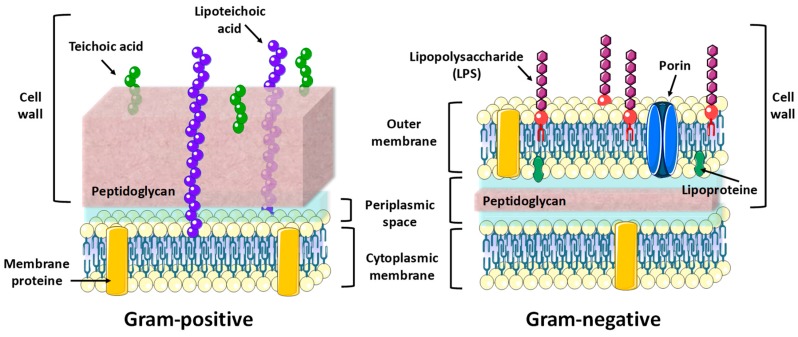
Comparison between structures and cell wall components of G^+^ and G^−^ bacteria. Build upon the material available in the website https://smart.servier.com.

One of the main challenges in nanomedicine is being able to achieve a selective treatment that allows us to act exclusively on the target area, without affecting healthy tissues, thus reducing the side effects of the drugs. In cancer, the enhanced permeability and retention effect (EPR), also called “passive targeting” is very common [81]. Despite the fact that certain studies suggest there is also a presence of this effect in bacterial infection [82,83], its role is not as relevant or as efficient when concentrating the nanoparticles in the vicinity of the infection. Therefore, the existence of an “active targeting” is even more necessary, as it allows us to direct the particles to the correct place. In addition, intracellular infections require a great specificity of treatment [84], as the bacteria can outwit the immune system and resist the macrophage-mediated killing mechanism, by surviving in its interior. In order to increase efficiency and selectivity of the antibiotic loaded nanoparticles, and to reduce the amount and frequency of the treatment, the surface of the MSNPs can be decorated with molecules that target bacteria, but that do not recognize the human host cells. The first difference between bacterial and human cells is that bacteria usually have a cell wall. The bacterial cell wall is a resistant and flexible layer that participates in the growth of the cell, and that allows it to withstand the osmotic force. It is composed partly of peptidoglycan and other glicolipids, exclusive of bacteria. The fact that human cells do not contain the same components means that these exclusive bacteria elements are the most important targets in bacteria [49,85,86]. Moreover, these components are so exclusive that they even differ, depending on the type of bacteria. Determined by the structure of its cell wall, bacteria are divided into two large groups: Gram-positive (G^+^) and Gram-negative (G^−^) (Figure 4). 

G^+^ bacteria are surrounded by a double layer composed of the cytoplasmic membrane and a thick layer of peptidoglycan containing teichoic acids. In the case of G^−^ bacteria, the protection is triple: the cytoplasmic membrane, a thin peptidoglycan layer, and an extra membrane called the outer membrane. In fact, this outer membrane is the reason of the greater resistance of G^−^ bacteria against antimicrobial agents and antibodies. Instead of teichoic acids in the surface of the outer membrane, we find lipopolysaccharides as an exclusive component of G^−^ bacteria. Therefore, by choosing the appropriate targeting molecule, it is possible not only to distinguish between human cells and bacteria, but to direct the nanoparticles to a specific type of bacteria. For instance teichoic acid antibodies or vancomycin can be used to target teichoic acids or peptidoglycan respectively in G^+^ bacteria and polymyxin can be used for selective targeting of the lipopolysaccharides of G^−^ bacteria [87,88,89].

**Figure 5:**
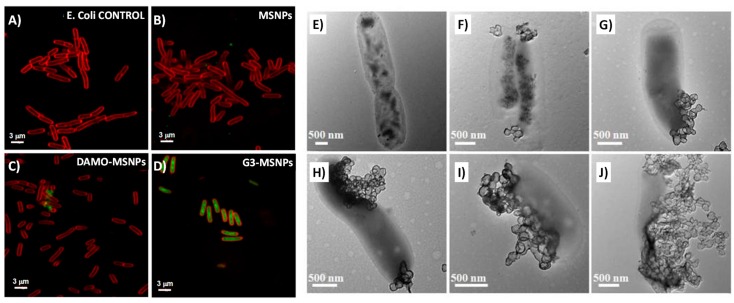
Left side: Confocal microscopy images of *E. coli* bacteria after treatment with (**A**) media, (**B**) pristine MSNPs, (**C**) *N*-(2-aminoethyl)-3-aminopropyltrimethoxysilane (DAMO)-MSNPs, and (**D**) G3-MSNPs at 10 mg/mL with an incubation time of 90 min. The *E. coli* cell membrane was stained with FM4-64FX (red), and the MSNPs were previously functionalized with fluorescein (green) (adapted with permission from [91], copyright Elsevier Ltd., 2017). Right side: Transmission electron microscopy (TEM) images of mycobacteria treated with Tre-hollow oblate mesoporous silica nanoparticles (HOMSNPs) loaded with isoniazid (INH) for 0 h (**E**), 0.5 h (**F**), 1 h (**G**), 2 h (**H**), 4 h (**I**), and 8 h (**J**) (adapted with permission from [41], copyright Wiley 2015).

Several studies show that for internalization, both types of bacteria appear to favor the presence of positive charges on the surface of nanoparticles [90]. M. Vallet-Regí et al. designed a novel nanovehicle, which was able to penetrate the cell wall of *E. coli* G^−^ bacteria, due to the presence of policationic dendrimers on its surface [91]. Confocal microscopy experiments have shown that the functionalized nanoparticles have an enhanced degree internalization compared with the pristine ones (Figure 5, left side). The sample loaded with levofloxacin also demonstrated that it has a great antibacterial efficacy. It was also observed that the antibiotic power of Levo and the disruption capacity of the policationic dendrimer have a synergistic effect. 

A similar study against *L. monocytogenes* G^+^ was carried out by J. M. Barat et al [92]. Equivalent results were obtained when studying the effect of anchoring polyamines on the surface of MSNPs. Bacteria have some processes such as stress responses, virulence, etc., that are regulated by a two-component system, and that do not exist in human cells. As an example, histidine kinase autophosphorylation inhibitors (HKAIs) are efficient bactericidal agents, but they do not affect humans. Although in theory they should affect all bacteria, HKAIs have proven to kill G^+^ bacteria, but they have almost no effect on G^−^. This is probably as a result of the composition of the lipopolysaccharides that are present on the G^−^ outer membrane that provide them with a great impermeability of hydrophobic antibiotics. To overcome this limitation, J. Wells et al. proposed the idea of capping HKAI-loaded MCM-41 NPs with ε-poly-l-lysine cationic polymer (ε-pLys) [93]. Results demonstrated that the positive charge of the capped NPs gave rise to a bactericidal effect on the G^−^, which was comparable to that shown for the free HKAIs against G^+^ bacteria. Another difficulty that compromises the effectiveness of nanomedicine is the immune system, specifically the action of macrophages that tend to eliminate everything that is alien to the organism, including NPs [94]. To prevent this from happening, H. Wang et al. designed vancomycin-modified MCM-41 NPs that selectively target and kill G^+^ bacteria over macrophage-like cells [95]. Vancomycin (Van) fulfills a double functionality: (i) it inhibits the normal development of the cell wall, favoring bacterial death and (ii) it targets G^+^ through specific hydrogen bonding interactions with the terminal d-alanyl-d-alanine moieties that are presented by this bacteria. Special mention should be given to the *Mycobacterium* genus, which falls within the G^+^ group, even though from an empirical point of view, they do not seem to be from this category, as their walls do not retain the dye responsible for their name. This is because the *Mycobacterium* cell wall is rich in mycolic acids, which gives it great strength. This unusual amount of mycolic acids has been used by M. Yan et al. as a distinguishing element to act precisely on this type of G^+^ bacteria [41,96]. First, they synthesized hollow oblate mesoporous silica nanoparticles (HOMSNPs) and decorated them with trehalose (Tre) as a targeting molecule, and then loaded them with isoniazid (INH, an antimycobacterial drug that inhibits the synthesis of mycolic acid) [97]. Trehalose is essential for mycobacteria, because it is one of the components that constitutes the mycolic acids and that participates in several transport processes through the cell wall [98]. As can be seen in Figure 5 (right side), after only 30 min of treatment the integrity of the mycobacteria appeared to be highly engaged. To test the targeting selectivity of Tre, two different experiments were performed: one substituting Tre for mannose as a control against *Mycobacterium smegmatis*; the second one exposed Tre-NPs to two different strains of G^−^ *E. coli* and G^+^ *S. epidermidis* bacteria. Both internalization and death were either non-existent, or much lower than that obtained for Tre-HOMSNPs in *M. smegmatis*. All the targeted MSNPs cited are summarized and referenced in Table 2.

### 2.2. Stimulus-Responsiveness

Despite the fact that MSM have demonstrated they are good as drug delivery agents, often, these nanocarriers have also shown the premature release of cargo molecules. However, as mentioned above, by being able to functionalize the surface of silica materials, this allows us to incorporate polymers or other molecules in order to temporarily block the pores of the nanocarriers, thus achieving a well-controlled release of the therapeutic agents. These temporary gates are considered to be “smart”, since they only open after the application of a stimulus that can be both internal and external. Nowadays, it is a very popular approach to use stimulus-responsive MSNPs for the treatment of cancer [99,100,101]. Although the use of these smart particles to treat bacterial infection is not so widespread, we can find some examples of its application in literature [102]. 

Several studies have demonstrated that bacterial infections are related to a decrease in the environmental pH, in anaerobic conditions [103,104]. If the multiplication process of the bacteria is strongly active during infection, it can result in the depletion of oxygen resources. This forces bacteria to obtain energy through an anaerobic metabolism, which, during the process, produces organic acids. Based on that, studies on the release of antibiotics from coated-MSNPs have been carried out according to the pH [105]. C-H. Lee et al. designed a silver−indole-3 acetic acid complex (IAAH) based on IBN-4 silica nanoparticles, which was able to release Ag ions under acidic conditions [106]. Apart from the well-known antimicrobial activity of the silver ions [107,108], a few years ago, it was also reported that Ag^+^ can generate ROS, which induce membrane damage and provide the sought-after bactericidal capacity [109]. The efficacy of these Ag-IAAH-MSNPs was studied against different types of bacteria; both in the planktonic state once the biofilm was formed, and even in vivo, obtaining, in all cases, very promising results, which demonstrate the potential of this system for the treatment of bacterial infection.

**Figure 6:**
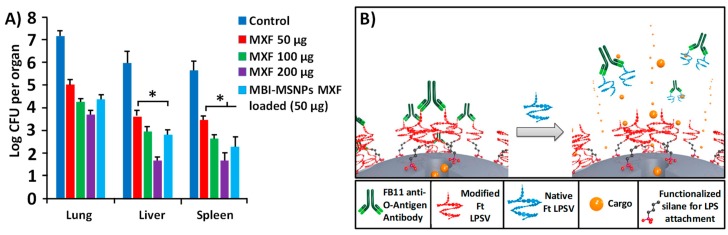
(**A**) In vivo efficacy of 1-methyl-1H-benzimidazole (MBI)-MSNPs loaded with moxifloxacin (MXF), assessed by an assay of *F. tularensis* burden in the mouse organs. Post-infection, mice were treated with free MXF or with MXF-loaded MBI-MSNPs by tail vein injection on days 1, 3, and 5. Mice were euthanized one day after the last dose of treatment (day 6) to enumerate the bacterial numbers in the lung, liver, and spleen (adapted with permission from [110], copyright American Chemical Society 2015). (**B**) Schematic representation of the triggered release of cargo due to a competitive displacement of the antibody (green) that caps the pore by naturally occurring *F. tularensis* lipopolysaccharide (LPS) (blue) (adapted with permission from [111], copyright American Chemical Society 2017).

In 2015 J. Zink et al. proposed using pH sensitive MSNPs, loaded with moxifloxacin (MXF) to treat the *F. tularensis* infection [110]. The opening mechanism was based on a two component system; 1-methyl-1H-benzimidazole (MBI) stalk covalently attached to the MSNP’s surface, which at a physiological pH holds the cyclodextrin (CD) caps during hydrophobic interactions. After being internalized by the cells, at the acidic pH of the endosomes the stalk is protonated and the binding affinity between the two elements drastically decreases, leading to the cargo release. Due to how MSNPs are avidly internalized by macrophages, the use of this device for tularemia treatment is very advantageous and such cells are one of the primary targets for *F. tularensis*. The in vivo efficacy of the device was then tested in a mouse model, of pneumonic tularemia. The results showed that without treatment, the mice rapidly feared signs of infection, while those treated had a healthier appearance and the bacterial presence in their organs was much lower (Figure 6A).

Other than the pH, other stimulus has also been used for the controlled release of drugs in the treatment of bacterial infection. T. Tang conceived a MCM-41 based nanodevice that is able to release their cargo in the presence of bacterial toxins [56]. After loading it with gentamicin (Gen), to avoid premature release, the MSNPs were coated with a liposome bilayer (L). However, at infection sites the presence of bacterial phosphatases, lipases, and phospholipases degraded the liposomes, allowing for the controlled release of Gen. In addition, to achieve an even more effective treatments, the liposome bilayer was functionalized with UBI29−41 (U), an ubiquicidin peptide for bacteria targeting. Having achieved good results against bacteria, the system was then studied in vivo in *S. aureus*-infected mice. Data demonstrates the excellent target efficiency of UL-MSNPs, as well as their antibiotic activity, compared with the Gen treatment.

According to the authors’ opinions, and despite being a preliminary study, a potential effective approach in terms of controlled drug release is that adopted by J. Zink. In this system, which combines targeting and the stimulus-response sensitivity, the binding to the bacterium itself is what triggers the release of the drug [111], thus increasing the selectivity of the treatment. The pores of MCM-41 MSNPs were blocked with FB11, an antibody for lipopolysaccharide (LPS) that is present in the *Francisella tularensis* (Ft) bacteria wall. To link the caps, a derivative (with a reduced affinity) of the O-antigen of Ft LPS (modified Ft LPS) was immobilized onto the surface of the nanoparticles. Thereby, FB11 targets the nanoparticles towards the bacteria, and once on its surface, the presence of the native antigen Ft LPS, which has a greater affinity for the antibody, displaces the modified one. This causes the pores to open, and therefore release the load. The authors used a dye to incubate the Ft and *Francisella novocida* (Fn) with the nanoparticles and compared the fluorescence levels after 1 h. They observed that the intensity detected in Ft was 100 a.u., while the value for Fn was only 20 a.u, which provides large selectivity of the nanoplatform in vitro. All the stimuli-responsive MSNPs cited are summarized and referenced in Table 3.

## 3. Effect on Biofilms

As previously mentioned, a biofilm is an assembly of microorganisms that grow strongly connected to a surface and are protected by a matrix of primarily polysaccharide material [112]. The complexity of the biofilm can be variable, as it can be a comprise of a single microbial species, or a combination of different ones [113]. Regardless of the species, the formation of the biofilm is the result of a sequence of phenomena [114]. First, the bacteria is adhered to a surface, then there is the proliferation and differentiation stage, during which the bacteria begin to secrete an exopolysaccharide. This constitutes the extracellular matrix and culminates with dispersion, where some bacteria leave the initial focus of infection in order to colonize new surfaces [115]. The adhesion of bacteria to a surface as a means of protection is the most accepted explanation for biofilm formation. The main challenge of biofilm formation is that bacteria acquire a greater tolerance to both the antibiotics and the response of the immune system. While in the planktonic state, all bacteria are exposed to the presence of biocides or immune cells, in the biofilm, bacteria are structurally organized in such a way that only the external ones, which are also protected by the polysaccharide matrix, receive this action [113]. Although the polysaccharide matrix acts as a first physical barrier, there are other reasons for why biofilms acquire greater tolerance to antibiotics [116]. These include: (i) the presence of other components in the matrix such as bacterial and host DNA and bacterial proteins that bacteria can use to their own benefit, for instance, increasing the shielding capacity of the matrix even more [117]; (ii) a population of bacteria with a varied physiology, especially in terms of growth states, that present different sensitivities to the same treatment [118]; (iii) the development of specific protective tools such as efflux pumps [119]; (iv) the synthesis of antibiotic-degrading enzymes [120]; (v) the establishment of a bacteria–bacteria communication, also known as quorum sensing [121]; and (vi) an active adaptation of the microenviromental limitations [122]. 

In addition to all these defense mechanisms, even after being damaged, the biofilm has the capacity to reconstruct again if it was not entirely eliminated [123]. Due to its complexity and the serious consequences of its appearance, it is not difficult to understand why in the last few years the number of studies that are focused on both preventing and treating bacterial biofilm formation has increased considerably. 

### 3.1. Prevention of Biofilm Formation

Since the first phase of biofilm formation is bacterial adhesion, much effort has been focused on understanding the factors that affect this process. The surfaces to which the bacteria can adhere are very varied, including wounds, implantable devices, and even one another [122]. However, biofilms are produced preferentially in certain parts of the body (such as the urinary tract [124,125], mitral valves [126], lungs [127], or the middle-ear (especially in children) [128,129]) and implanted devices. Although infections in our own tissues are difficult to prevent, it is possible to minimize the number of infections that are associated with implants. Nowadays medical aids allow us to implant diverse materials into the organism to improve the quality of life of the patients [127,130]. Although these materials have been made biocompatible and they therefore do not cause adverse reactions or rejections by the immune system, the introduction of a foreign element into the body generates an optimal niche for the formation of a biofilm. Some of the most common implants for where the formation of the biofilm takes place are: dental implants, heart valve implants or pacemakers, and other implants of the hip [131,132,133]. The appearance of infections are also frequent after the use of temporary or partial implantation devices, such as catheters.

**Figure 7:**
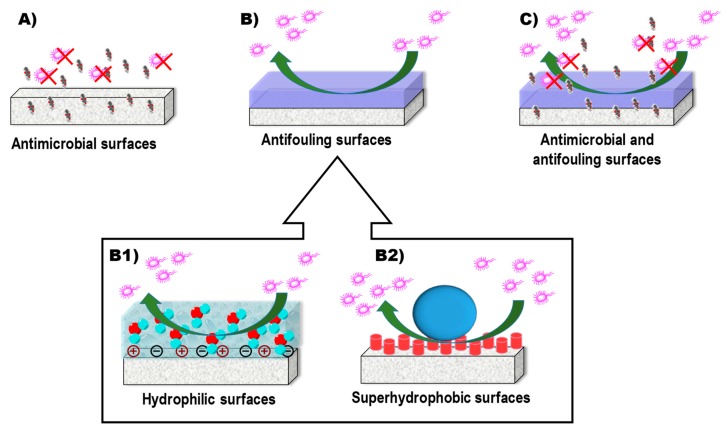
Current strategies for developing biofilm-resistant biomaterials. (**A**) Antimicrobial-loaded/functionalized materials; (**B**) antifouling materials due to (**B1**) hydrophilic or (**B2**) superhydrophobic surfaces; (**C**) combining antifouling and antimicrobial effects in the same material.

To avoid that, the scientific interest aimed at developing biomaterials that are less susceptible or even resistant to bacterial infections has significantly increased in recent years [134,135,136,137]. So far, different strategies have been used for this purpose (Figure 7): (i) loading/functionalizing the material with antimicrobial substances [138], (ii) biomaterial surface modification to give anti-fouling capabilities [139,140,141], (iii) combining antifouling and antimicrobial effects in the same coating [142,143]. 

If we focus on the use of porous silica materials as a way to prevent the formation of the bacterial biofilm, we can highlight the following advances, which are also summarized in Table 4. 

Several studies have demonstrated that loading an antimicrobial agent onto silica pores can reduce biofilm formation, since the release of the drug eliminates the surrounding bacteria and their adhesion to the surface of the material [70,77,144,145,146,147]. Thus, these materials could be good candidates for covering other surfaces that are more susceptible to being colonized by bacteria. M. C. Chifiriuc et al. used cephalosporin (Ceph)-loaded MCM-48 MSNPs as an implant coating to prevent microbial biofilm formation [148]. They observed that for the coated samples, the sustained release of Ceph rested on a significant reduction of *E. coli* biofilm formation, especially during the first 24 h. P. Behrens et al. coated the implant surfaces with a layer of ciprofloxacin (Cip)-loaded mesoporous silica. They then investigated the effect of the sulfonic acid functionalization of the pores on the drug-loading process [149]. In addition, the authors managed to extend the Cip release time by up to 60 days by adding successive silica derivative layers. When tested against *P. aeruginosa* bacteria, the sulfonate-functionalized material, which was loaded with Cip and then modified with bis(trimethoxysilyl)hexane, showed the best anti-biofilm capacity. Recently, A. Yu et al. performed a similar study, but loaded gentamicin (Gen) onto the silica pores, and used a Nafion layer to slow down the Gen release and prolong its antibacterial activity for more than two months [150]. The use of dental implants is a very common practice in our days. However, the appearance of infection after surgery still occurs at a significant rate. Due to its lightness, its resistance, and especially its biocompatibility, titanium is one of the most commonly used materials in implants of all types, including dental. However, bacteria easily adhere to their surface, causing implant failure. To avoid this, several groups have developed new composite coatings that combine other materials (usually polymers) with drug-loaded silica NPs [151,152]. In all studies, these coatings demonstrate that they are able to provide a strong antimicrobial capacity to the titanium surface, both reducing the biofilm formation and killing the bacteria near the surface. Poly(methyl methacrylate) (PMMA) is another material widely used in dental clinics, which has been modified with nanomaterials to improve its antibacterial properties [153]. R. Tan et al. [154] and H-H. Lee et al. [155] studied the effect of adding drug loaded silica NPs to PMMA. Both observed a clear enhanced antibacterial effect compared with the pure PMMA, while the mechanical properties of the material were negligibly affected. Other cement materials combined with silica NPs have also demonstrated great results in avoiding biofilm and caries formation [156,157]. 

**Figure 8:**
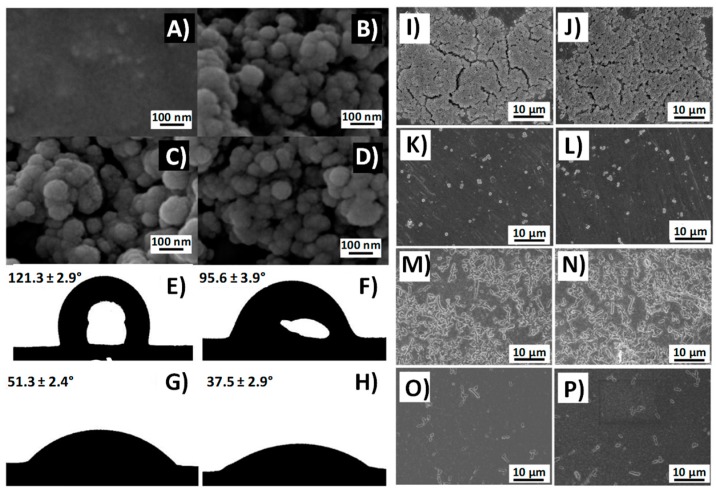
Scanning electron microscopy (SEM) images of (**A**) Silicone film (Si), (**B**) MSNPs coated Si (MSNPs-Si), (**C**) Agarose-incorporated MSNPs-Si (AMSNPs-Si), and (**D**) Heparin-loaded AMSNPs-Si, respectively (HAMSNPs-Si). The static water contact angle of (**E**) Si, (**F**) MSNPs-Si, (**G**) AMSNPs-Si, and (**H**) AHMSNPs-Si. SEM images of (**I**–**L**) *S. aureus* and (**M**–**P**) *E. coli* cultured on (**I**,**M**) Si, (**J**,**N**) MSNPs-Si, (**K**,**O**) AMSNPs-Si, and (**L**,**P**) AHMSNPs-Si, respectively (adapted with permission from [161], copyright American Chemical Society 2017).

It is well known that bacteria and other microorganisms tend to adhere to a surface due to the presence of hydrophobic interactions [158]. Therefore, reducing these interactions by making the surface of the material more hydrophilic reduces the probability of biofilm formation. It is considered that the non-stick ability of hydrophilic materials is closely related to a hydration layer formed close to the surface [159]. Its presence may hinder the approximation of bacteria to the material surface and therefore, decreases the likelihood of its irreversible adhesion and the formation of the biofilm. Agarose; an hydrophilic polymer [160] derived from agar, was incorporated into silica NPs and used as an antifouling coating for silicone biomaterials. J. Shen et al. observed that loading agarose and heparin into the same system allowed them to achieve a coating with a double functionality: antifouling and anticoagulant [161]. The results showed a decrease in bacterial adhesion, due to the presence of agarose and an absence of platelets, due to the anticoagulant action of the released heparin (Figure 8). 

Another strategy that was used to design hydrophilic materials involves the synthesis of zwitterionic surfaces. Zwitterionic materials are those that present the same number of positive and negative charges on its surface, thus preserving the electrical neutrality [162]. When talking about obtaining zwitterionic silica materials, there are several strategies that can be summarized: (i) functionalization with zwitterionic elements, or (ii) anchoring mixtures of molecules whose net average charge is zero [55]. M. Vallet-Regí et al. designed the first porous silica material with zwitterionic character at physiological pH [143]. The surface was functionalized with (*N*-(2-aminoethyl)-3-aminopropyltrimethoxysilane) (DAMO). This alkoxysilane presents primary and secondary amines that are positively charged at pH 7.4, which, in combination with the negative charge characteristics of the silanols, provided the desired zwitterionic character. In addition, the pores were loaded with cephalexin to achieve a sustained release that killed the surrounding bacteria. Therefore, they took advantage of the properties of the SBA-15 to design a new material with dual antibacterial capability against *S. aureus*. 

**Figure 9:**
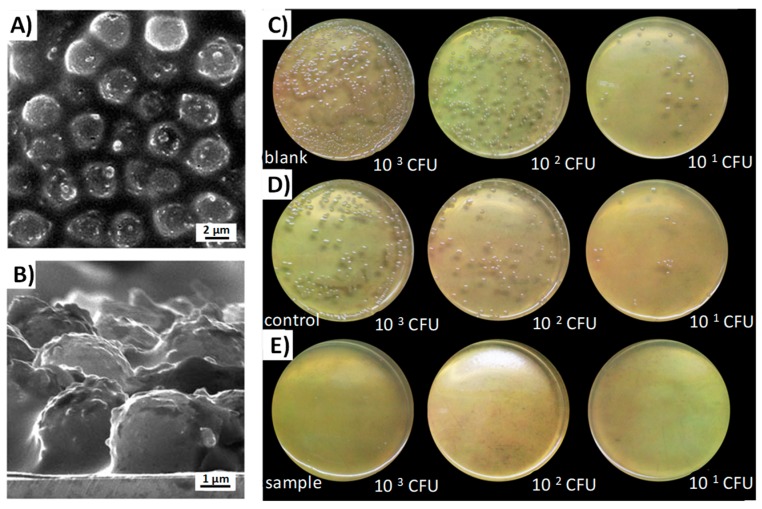
Scanning electron microscopy (SEM) images of (**A**) the film made of mesoporous silica microcapsules with AgNPs supported onto the interior walls, incorporated onto the hydrophobic fluoro-silicone resin (MSMAs/FSR film); (**B**) the cross-sectional structure of the film. Antibacterial activities of various samples against *E. coli* in different initial bacterial concentrations: (**C**) the blank (without film); (**D**) the control (with the pure fluoro-silicone resin (FSR) film); (**E**) the sample (with the MSMAs/FSR film) (adapted with permission from [171], copyright The Royal Society of Chemistry 2014).

We have mentioned that while hydrophilic surfaces repel bacterial adhesion, hydrophobic surfaces favor it. However, numerous studies show that achieving an extreme hydrophobic character, known as “superhydrophobicity”, re-equips the materials with antifouling properties [163,164,165,166]. A superhydrophobic material is one that is very difficult to wet [167]. The lotus leaf is the most representative example, as the contact angle of water is over 150° [168]. It is believed that its “unwettability” is due to micro/nanosized roughness, and to a hierarchical structure [169]. Since the size of the bacteria is in the same order of magnitude, the effects of nanoscale features that also affect surface energy, have shown that they influence the binding processes [164]. Therefore, by using superhydrophobic materials, it is possible to reduce the bacteria–surface adhesion forces, hindering their union, facilitating their removal, and limiting biofilm formation [170]. C. Wu et al. synthesized Ag-supported mesoporous silica microcapsules and incorporated them into a hydrophobic fluoro-silicone resin, forming a rougher nanostructure [171]. Results showed that this new material reached an antibacterial rate of 99.3%, because of the combination of action of released silver ions and a superhydrophobic surface (Figure 9). 

### 3.2. Effect on the Formed Biofilms

Without any doubt, the best option is to try to avoid biofilm formation with any of the aforementioned strategies or materials. However, if it has already been formed, and taking into account how difficult it is to completely eliminate a biofilm, nowadays, the most effective procedure is to eliminate the infected area or device [172,173]. However, this is a great inconvenience for patients, and carries a high hospitalization cost, not to mention that in some cases, it is not possible to remove [174]. As far as we are concerned, the most promising alternative to surgery involves the disruption of the biofilm to make it more vulnerable to the action of the antibiotics and the immune system. Recently, some groups have reported the use of small molecules [175], metal nanoparticles [176], bacteriophages [177], or enzymatic lysis [178] as methods for destroying the biofilm matrix backbone. Investigations have also been carried out into the efficacy of targeting [179] biofilms by the modification of the nanoparticles surface, by covalently attached antibodies [180], dendrimers [181,182], or lectins [183]. 

**Figure 10:**
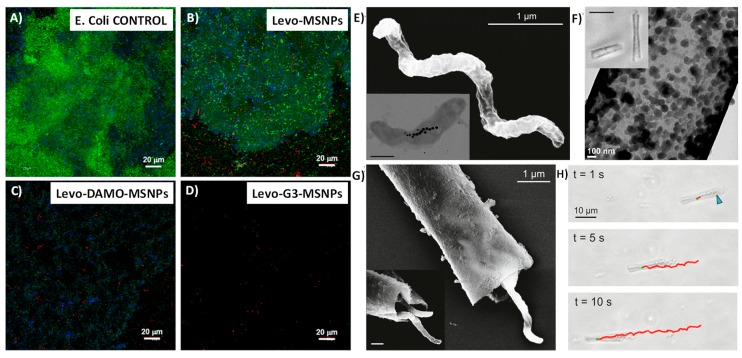
(**A–D**) Confocal microscopy study of the antimicrobial activity of the Levo-loaded MSNPs materials onto Gram-negative *E. coli* biofilm. Live bacteria are stained in green, dead bacteria in red, and the matrix biofilm in blue (adapted with permission from [91], copyright Elsevier Ltd., 2017). (**E**–**H**) Characterized bacteria-driven biohybrid micro-swimmers. (**E**) SEM image of MSR-1 bacterium. Inset displays TEM of MSR-1 and the internal magnetosome chain. Inset scale bar = 500 nm. (**F**) TEM image of pores in MSMi. Inset displays bright field microscopy images of long and short MSMi. Inset scale bar = 10 μm. (**G**) SEM of MSR-1 cells captured within a microtube. Inset displays increased magnification of bacteria in the tube. Inset scale bar = 500 nm. (**H**) Bright field microscopy images of MSR-1-powered biohybrids swimming. Blue arrow indicates location of MSR-1 inside the microtube. Red track indicates the trajectory of the biohybrid (adapted with permission from [186], copyright American Chemical Society 2017).

In this sense, the use of MSM as DDS that are capable of disrupting and penetrating into the biofilm and once there releasing their cargo, to increase their antimicrobial efficiency, has great potential. These type of studies have recently begun, and therefore, the number of publications on this subject is limited. Q. Ye et al. reported their use of rod shaped hollow MSNPs loaded with lysozyme (HMSNPs-LP@Lys) to act inside the biofilm [184]. A clear increase in bacterial killing capability was observed for HMSNPs-LP@Lys compared with that obtained for the free drug. Additionally, the biocompatibility of the device was tested towards mammal cells, with the achievement of around 80% survival. Several studies performed by M. Vallet-Regí et al. demonstrated that the presence of positive charges on the surface of the silica NPs not only favors the affinity and disruption of the bacterial wall, but also of the feared biofilm. Thus, MCM-41 silica NPs were loaded with levofloxacin (Levo, a wide range fluoroquinolone antibiotic) and functionalized with the amino group containing molecule *N*-(2-aminoethyl)-3-aminopropyltrimethoxysilane (DAMO) [185]. To test the targeting effect, grown biofilms were exposed to both functionalized and non-functionalized particles. Confocal images showed that while DAMO decorated NPs (D-MCM-41) internalized inside the biofilm, the bare ones remained mostly on the surface. When the biofilm were exposed to the loaded nanoparticles, a clear reduction of the biofilm was observed for that treated with Levo@D-MCM-41. However, for those treated with Levo@MCM-41, a remaining layer persisted. In another study, the MCM-41 were functionalized with a poly(propyleneimine) third-generation dendrimer (G3), richer in amino groups [91]. In this case after adding Levo@G3-MCM-41 the biofilm was totally destroyed (Figure 10A–D). 

In 2017, S. Sánchez et al. proposed the use of bacteria as a new method of transport for mesoporous silica microtubes (MSMi) into the biofilm [186]. *Magnetosopirrillum gryphiswalense* (MSR-1) are nonpathogenic magnetotactic bacteria (Figure 10E) that are capable of being guided under the action of low magnetic fields. MSMi (Figure 10F) with the appropriate size to harbor MSR-1 inside (Figure 10G) were synthesized and loaded with ciprofloxacin, an antibiotic that is soluble only under acidic conditions. Results demonstrated that they were able to guide the MSR-1/MSMi biohybrid to the biofilm, and to force its penetration by magnetic action (Figure 10H), allowing the release of the cargo by the presence of acidic conditions, once internalized. All the MSNPs for the treatment of the formed biofilm cited are summarized and referenced in Table 5.

## 4. Drawbacks

Despite the numerous advantages that the use of MSM as DDS entails for the treatment and prevention of bacterial infection, the use of these materials also has certain disadvantages. 

Some of them are general for all nanoparticles, regardless of their nature. One of them is related to its aggregation in vivo, while in vitro studies offer good results, the complexity of the medium in vivo can affect the stability of the particles in suspension, altering its effectiveness. Another one is the reproducibility, which, since it is not perfect, requires that each batch must be individually characterized and tested. Perhaps because of this, similar particles provide contradictory results in different studies. In addition, each modification made in the nanoparticle can have potential repercussions on cells and bacteria, so that it is considered to be a different system that has to be re-characterized, greatly increasing the number of experiments, and delaying the time necessary for it to be used under clinical conditions. In addition, the more complex the system, the more difficult it is to understand its method of action, because it can have several antibiotic/toxic effects at the same time.

Specifically for MSM, although it has been seen that they are biocompatible, some studies suggest that their use can produce hemolysis, because of the interaction of silanol groups with the phospholipids present in the membrane of red blood cells. However, more studies are needed to know the real impact of these materials on the body in the long term.

## 5. Conclusions and Future Outlook

After analyzing this bibliographic work, it is clear that drug delivery systems based on porous silica materials have a great potential in the treatment of bacterial infection. Although we have come a long way, there is still a lot of research to be done since certain aspects, such as stimulus–response systems or biofilm targeting, have only just begun to be studied. As stated above, so far, there is no universal treatment that is perfect on its own. However, a great advantage of MSM are their ability to be combined with other materials, thus achieving new and improved properties, and therefore a multifunctional applications. Thus, as mentioned in the title, the authors consider that mesoporous silica materials could be the definitive solution to bacterial infection, since they can attack it via numerous routes, not only those based on drug delivery that have been widely described in this manuscript, but also those that can be used for detection, as adjuvants, etc. 

We believe it is necessary to carry out further development of new antibacterial MSM, paying particular attention to their functionalization with specific biomolecules (e.g., antibodies, biomarkers etc.) for controlled specific targeting of pathogenic bacteria. This is particularly of great importance in medicine, as one major drawback of many current therapies is the problem of getting the drug into the site of interest. In addition, MSM present an excellent substrate and matrix for the development of new drug delivery systems with so-called “on demand” drug release. This could be achieved by loading pores with selected antibacterial drugs, and then by closing pores with appropriate “nano-plugs”, which could then be opened using light (e.g., quantum dots or large dye molecules), chemical (e.g., pH dependent cleavage) or magnetic (e.g., magnetic nanoparticles) initiation. This area is still to be developed; therefore, further research and the design of new materials and approaches for MSM preparation and modification will be highly important for future applications of these materials in biology and medicine. However, all these efforts will be in vain, unless society begins to use the treatments more carefully and responsibly. Currently the use of nanoparticles seems to be an effective alternative to treat bacterial infection, but if used abusively, the bacteria could potentially develop defense mechanisms and resistance against them.

## Figures and Tables

**Table 1:** Mesoporous Silica Materials in the Treatment of Planktonic Bacteria.

Type of Device ^a^	Type of Bacteria ^b^	Drug Loaded (*w*/*w* %) ^c^	Ref.
MCM-41 MSNPs	*E. coli*	Tetracycline (17.7–18.7%)	[67]
MCM-41 (MSNPs, N-MSNPs, C-MSNPs)	*E. coli, A. baumannii P. aeruginosa*	Polymyxin B (11.3–34.7%)	[69]
MCM-48 MSNPs	*S. aureus*, *E. coli*, *C. albicans*	EUC (7%), ORA (8%), CIN (41%) essentials oils	[70]
DMSNPs	*E. coli*	Lysozyme (3.47–24.4%)	[72]
biodegradable silica xerogel	*S. enterica*	Gentamicin (31%)	[73]
MCM-41 Ag@MSNPs	*E. coli, B. anthracis*	Ag nanocrystals (-)	[76]
Ag/DMSNPs	*S. aureus*, *E. coli*	Chlorhexidine (10.6%)	[68]
SBA-15 Ag/MSNPs	*S. aureus*	Peracetic acid (5.3%)	[77]
MCM-41 (MSNPs-SB-Cu, MSNPs-SB-Ni)	*E. coli*, *P. aeruginosa*, *S. aureus*, *B. subtilis*	Gentamicin (-)	[78]
MCM-41 Si-Ti-Sv	*E. coli, K. pneumoniae, M. morganii, P. mirabilis, E. faecalis*	Izohidrafural (6.8–30%)	[79]
DMSNPs@C-dots/RB	*E. coli*	Ampicillin (18.3%)	[80]

**^a^** MCM-41 MSNPs: Mesoporous silica nanoparticles with hexagonal mesoporous structure; N-MSNPs: aminated MSNPs; C-MSNPs: carboxylated MSNPs; MCM-48 MSNPs: MSNPs with cubic mesoporous structures; DMSNPs: MSNPs with a dendritic structure of pores; Ag@MSNPs: Nanoparticles with a silver core and coated with mesoporous silica; Ag/DMSNPs: DMSNPs decorated with silver NPs; SBA-15 Ag/MSNPs: Mesoporous silica nanoparticles with hexagonal mesoporous structures and microporous connections decorated with silver NPs; DMSNPs-SB-Cu: DMSNPs-supported copper, MSNPs-SB-Ni: MSNPs-supported nickel; Si-Ti-Sv: Silica–titanium sieves; MSNPs@C-dots/RB: carbon dots and Rose Bengal-embedded mesoporous silica nanoparticles. **^b^** *E. Coli*: *Escherichia coli*; *A. baumannii*: *Acinetobacter baumannii*; *P. aeruginosa*: *Pseudomonas aeruginosa*; *S. aureus*: *Staphylococcus aureus*; *C. albicans*: *Candida albicans*; *S. enterica*: *Salmonella enterica*; *B. anthracis*: *Bacillus anthracis*; *B. subtilis*: *Bacillus subtilis*; *K. pneumoniae*: *Klebsiella pneumoniae*; *M. morganii*: *Morganella morganii*; *P. mirabilis*: *Proteus mirabilis*; *E. faecalis*: *Enterococcus faecalis*. **^c^** EUC: eucalyptus; ORA: orange; CIN: cinnamon.

**Table 2:** Targeted Mesoporous Silica Nanoparticles for the Treatment of Planktonic Bacteria.

Targeting Molecule ^a^	Type of Bacteria ^b^	Drug Loaded (*w*/*w* %) ^c^	Type of Device ^d^	Ref.
Gram negative				
G3	*E. coli*	Levofloxacin (3.2–7.8%)	MCM-41 G3-MSNPs	[91]
ε-pLys	*E. coli*	HKAIs (0.5–13.8%)	MCM-41 ε-pLys-MSNPs	[93]
Gram positive				
Vancomicin	*S. aureus*	Vancomicin (anchored)(50%)	MCM-41 Van/MSNPs	[95]
Trehalose	*M. smegmatis*	INH (56.3–63.6%)	Tre-HOMSNs	[41]

**^a^** G3: polycationic dendrimer, poly(propyleneimine) dendrimer of the third generation; ε-pLys: ε-poly-l-lysine cationic polymer. **^b^** *E. coli*: *Escherichia coli*; *S. aureus*: *Staphylococcus aureus*; *M. smegmatis*: *Mycobacterium smegmatis*. **^c^** HKAIs: histidine kinase autophosphorylation inhibitors; INH: isoniazid; **^d^** MCM-41 G3-MSNPs: MCM-41 type MSNPs functionalized with G3; MCM-41 ε-pLys-MSNPs: MCM-41 type MSNPs functionalized with ε-pLys; MCM-41 Van-MSNPs: MCM-41 type MSNPs functionalized with vancomycin; Tre-HOMSNs: Trehalose-functionalized hollow oblate mesoporous silica nanoparticles.

**Table 3:** Stimuli-Responsive Mesoporous Silica Nanoparticles for the Treatment of Planktonic Bacteria.

Stimuli ^a^	Type of Bacteria ^b^	Drug Loaded (*w*/*w* %) ^c^	Type of Device ^d^	Ref.
pH	*E. coli*, *B. subtilis*, *S. aureus*, *S.epidermidis*	Ag^+^ (70%)	IBN-4 IAAH-MSNPs	[106]
pH	*F. tularensis*, *F. tularensis*-infected mice	Moxifloxacin (51.4%)	MCM-41 MBI-MSNPs	[110]
Bacterial toxins	*S. aureus*, *S. aureus*-infected mice	Gentamicin (25.6%)	MCM-41 UL-MSNPs	[56]
antigen Ft LPS	*F. tularensis, F. novocida*	Hoechst (-)	MCM-41 FB11-mFt LPS-MSNPs	[111]

**^a^** Ft LPS: LPS present in *F. tularensis* (Ft). **^b^** *E. coli*: *Escherichia coli*; *B. subtilis*: *Bacillus subtilis*; *S. aureus*: *Staphylococcus aureus*; *S. epidermidis*: *Staphylococcus epidermidis*; *F. tularensis*: *Francisella tularensis*. **^c^** IBN-4 IAAH-MSNPs: Silver–Indole-3 Acetic Acid Complex based on IBN-4 silica nanoparticles; MCM-41 MBI-MSNPs: 1-methyl-1H-benzimidazole functionalized MSNPs with MCM-41 structure; UL-MSNPs: MSNPs coated with a liposome bilayer and functionalized with the ubiquicidin peptide (UBI_29–41_); FB11-mFt LPS-MSNPs: MSNPs functionalized with an antibody for Ft LPS through a derivative of the O-antigen of Ft LPS.

**Table 4:** Mesoporous Silica Materials for Preventing Biofilm Formation.

Strategy	Type of Bacteria ^a^	Drug Loaded (*w*/*w* %) ^b^	Type of Device ^c^	Ref.
Antimicrobial effect	*S. aureus*, *E. coli*, *C. albicans*	EUC (7%), ORA (8%), CIN (41%) essentials oils	MCM-48 MSNPs	[70]
Antimicrobial effect	*S. aureus*	Peracetic acid (5.3%)	SBA-15 Ag/MSNPs	[77]
Antimicrobial effect	*S. aureus*, *S. epidermidis*	Vancomycin (−), Rifampin (−)	SBA-15 MSNPs	[144]
Antimicrobial effect	*E. coli*	Cephalosporin (−)	MCM-48 MSNPs	[148]
Antimicrobial effect	*P. aeruginosa*	Ciprofloxacin (2 µg·cm^−2^)	LMU-1 bth@MSNPs	[149]
Antimicrobial effect	*S. aureus*	Gentamicin (21.9%)	MCM-41 Nafion@MSNPs	[150]
Antimicrobial effect	*S. aureus*	Gentamicin (−)	Gel/SiO_2_-Gen NP-coated Ti	[151]
Antimicrobial effect	*A. actinomycetemcomitans*	Ag NPs (4.26%)	AgNP/NSC-coated Ti	[152]
Antimicrobial effect	*S. aureus*	Gentamicin (−)	SBA-15 PMMA/MSNPs	[154]
Antimicrobial effect	*C. albicans*, *S. oralis*	Amphotericin B (2.5–7.2%)	MCM-41 PMMA/MSNPs	[155]
Antimicrobial effect	*S. mutans*	EGCG (11.29%)	MCM-41 nHAp@MSNPs	[156]
Antimicrobial effect	*S. mutans*	Chlorhexidine (44.62%)	pMCM-41-GIC	[157]
Anti-fouling effect	*E. coli*, *S. aureus*	Heparine (5.7%)	AMSNPs-Si	[161]
Anti-fouling and antimicrobial effects	*S. aureus*	Cephalexin (1.2%)	SBA-15 DAMO-MSM	[143]
Anti-fouling and antimicrobial effects	*E. coli*	Ag NPs (20.34%)	MSMAs/FSR film	[171]

**^a^***S. aureus*: *Staphylococcus aureus*; *E. coli*: *Escherichia coli*; *C. albicans*: *Candida albicans*; *S. epidermidis*: *Staphylococcus epidermidis*; *P. aeruginosa*: *Pseudomonas aeruginosa*; *A. actinomycetemcomitans*: *Aggregatibacter actinomycetemcomitans*; *S. oralis*: *Streptococcus oralis*; *S. mutans*: *Streptococcus mutans*. **^b^** EUC: eucalyptus; ORA: orange; CIN: cinnamon; EGCG: epigallocatechin-3-gallate, a polyphenolic compound extracted from tea leaves that exhibits antibacterial activity. **^c^** SBA-15 Ag/MSNPs: Mesoporous silica nanoparticles with hexagonal mesoporous structures and microporous connections decorated with silver NPs; LMU-1 bth@MSNPs: bis(trimethoxysilyl)hexane (bth)-coated MSNPs with non-ordered pores; Nafion@MSNPs: Nafion-coated MSNPs; Gel/SiO_2_-Gen NP-coated Ti: titanium surface coated with a composite made of gelatin and “gentamicin-rich nuclei” silica nanoparticles; AgNP/NSC-coated Ti: titanium surface coated with a silica-based composite coating containing silver nanoparticles; SBA-15 PMMA/MSNPs: poly(methyl methacrylate) resin containing SBA-15 MSNPs; MCM-41 PMMA/MSNPs: poly(methyl methacrylate) resin containing MCM-41 MSNPs; MCM-41 nHAp@MSNPs: nanohydroxyapatite/mesoporous silica nanoparticles; pMSN-GIC: glass ionomer cement containing expanded-pore MCM-41 MSNPs; AMSNPs-Si: (agarose-loaded mesoporous silica nanoparticles) coating immobilized on silicone films; SBA-15 DAMO-MSM: SBA15 mesoporous silica material functionalized with (*N*-(2-aminoethyl)-3-aminopropyltrimethoxysilane); MSMAs/FSR film: film made of mesoporous silica microcapsules with AgNPs supported onto the interior walls incorporated onto the hydrophobic fluoro-silicone resin.

**Table 5:** Mesoporous Silica Nanoparticles for the Treatment of the Formed Biofilm.

Targeting Molecule ^a^	Type of Bacteria ^b^	Drug Loaded (*w*/*w* %)	Type of Device ^c^	Ref.
-	*E. coli*	Lysozyme (35%)	HMSNPs-LP	[184]
-	*S. aureus*	Ag^+^ (70%)	IBN-4 IAAH-MSNPs	[106]
DAMO	*E. coli*, *S. aureus*	Levofloxacin (3.2–5.0%)	MCM-41 DAMO-MSNPs	[185]
G3	*E. coli*	Levofloxacin (3.2–7.8%)	MCM-41 G3-MSNPs	[91]
MSR-1	*E. coli*	Ciprofloxacin (−)	MSR-1/MSMi	[186]

**^a^** DAMO: *N*-(2-aminoethyl)-3-aminopropyltrimethoxysilane; G3: poly(propyleneimine) third-generation dendrimer; MSR-1: *Magnetosopirrillum gryphiswalense*. **^b^** *E. coli*: *Escherichia coli*; *S. aureus*: *Staphylococcus aureus*; **^c^** HMSNPs-LP: rod shaped hollow MSNPs with large cone shaped pores; IBN-4 IAAH-MSNPs: Silver–ondole-3 acetic acid complex based on IBN-4 silica nanoparticles; MCM-41 DAMO-MSNPs: MCM-41 type MSNPs functionalized with DAMO; MCM-41 G3-MSNPs: MCM-41-type MSNPs functionalized with G3; MSR-1/MSMi: Mesoporous silica microtubes (MSMi) with MSR-1 inside.

## Abbreviations

*A. actinomycetemcomitans*	*Aggregatibacter actinomycetemcomitans*
*A. baumannii*	*Acinetobacter baumannii*
AFM	Atomic force microscopy
Ag/DMSNPs	DMSNPs decorated with silver NPs
Ag@MSNPs	Nanoparticles with silver core and coated with mesoporous silica
AMSNPs-Si	(Agarose-Loaded Mesoporous Silica Nanoparticles) Coating Immobilized on Silicone Films
*B. anthracis*	*Bacillus anthracis*
*B. subtilis*	*Bacillus subtilis*
bth	bis(trimethoxysilyl)hexane
*C. albicans*	*Candida albicans*
CD	Cyclodextrin
C-dots	Carbon dots
Ceph	Ccphalosporin
CFU	Colony Forming Units
CIN	cinnamon
Cip	Ciprofloxacin
C-MSNPs	carboxilated MSNPs
DAMO	*N*-(2-aminoethyl)-3-aminopropyltrimethoxysilane
DDS	Drug delivery systems
DMSNPs	Dendritic mesoporous silica nanoparticles
DMSNPs-SB-Cu	DMSNPs supported copper
*E. coli*	*Escherichia coli*
*E. faecalis*	*Enterococcus faecalis*
ECDC	European Centre for Disease Prevention and Control
EFSA	European Food Safety Authority
EGCG	Epigallocatechin-3-gallate
EPR	Enhanced permeability and retention
EUC	eucalyptus
FB11	Antibody for lipopolysaccharide (LPS) present in the *Francisella tularensis*
FB11-mFt LPS-MSNPs	MSNPs functionalized with an antibody for Ft LPS through a derivative of the O-antigen of Ft LPS
FM4-64FX	FM 4-64FX, fixable analog of FM 4-64 membrane stain
Fn	Francisella novocida
Ft LPS	lipopolysaccharide (LPS) present in *F. tularensis* (Ft)
Ft	*Francisella tularensis*
G^−^	Gram negative bacteria
G^+^	Gram positive bacteria
G3	polycationic dendrimer, poly(propyleneimine) dendrimer of third generation
Gel/SiO_2_-Gen NP coated Ti	titanium surface coated with a composite made of gelatin and “gentamicin-rich nuclei” silica nanoparticles
Gen	Gentamicin
HEK293	Human embryonic kidney 293 cells
HKAIs	Histidine kinase autophosphorylation inhibitors
HMSNPs-LP	rod shaped hollow MSNPs with large cone shaped pores
HOMSNPs	Hollow oblate mesoporous silica nanoparticles
IAAH	Indole-3 Acetic Acid Complex
IBN-3	Mesocellular foam
IBN-4 IAAH-MSNPs	Silver–Indole-3 Acetic Acid Complex based on IBN-4 silica nanoparticles
INH	Isoniazid
Izo	izohidrafural
*K. pneumoniae*	*Klebsiella pneumoniae*
Levo	Levofloxacin
LMU-1 bth@MSNPs	bis(trimethoxysilyl)hexane (bth) coated MSNPs with non-ordered pores
LMU-1	Disordered mesoporous structure
LPS	Lipopolysaccharide
LB	Luria-Bertani liquid medium
lys	Lysozyme
*M. morganii*	*Morganella morganii*
*M. smegmatis*	*Micobacterium smegmatis*
MBI	1-methyl-1H-benzimidazole
MCM-41 DAMO-MSNPs	MCM-41 type MSNPs functionalized with DAMO
MCM-41 G3-MSNPs	MCM-41 type MSNPs functionalized with G3
MCM-41 MBI-MSNPs	1-methyl-1H-benzimidazole functionalized MSNPs with MCM-41 structure
MCM-41 nHAp@MSNPs	nanohydroxyapatite/mesoporous silica nanoparticles
MCM-41 PMMA/MSNPs	Poly(methyl methacrylate) resine containing MCM-41 MSNPs
MCM-41 Van-MSNPs	MCM-41 type MSNPs functionalized with Vancomicin
MCM-41 ε-pLys-MSNPs	MCM-41 type MSNPs functionalized with ε-pLys
MCM-41	Mesoporous structure with a hexagonal pattern
MCM-48	Mesoporous structure with a cubic pattern
MIC	Minimun Inhibiory Concentration
MSM	Mesoporous silica materials
MSMAs/FSR film	Film made of mesoporous silica microcapsules with AgNPs supported onto the interior walls, incorporated onto the hydrophobic fluoro-silicone resin
MSMi	Mesoporous silica microtubes
MSNPs	Mesoporous silica nanoparticles, regardless of its shape
MSNPs@C-dots/RB	carbon dots and Rose Bengal embedded mesoporous silica nanoparticles
MSNPs-SB-Ni	MSNPs supported nickel
MSR-1/MSMi	Mesoporous silica microtubes (MSMi) with MSR-1 inside
MSR-1	Magnetosopirrillum gryphiswalense
MXF	Moxifloxacin
Nafion@MSNPs	Nafion coated MSNPs
nHAp@MSNPs	nanohydroxyapatite/mesoporous silica nanoparticles
NMs	Nanomaterials
N-MSNPs	aminated MSNPs
NPs	Nanoparticles
ORA	orange
*P. aeruginosa*	*Pseudomonas aeruginosa*
*P. mirabilis*	*Proteus mirabilis*
PMMA	Poly(methyl methacrylate)
pMSN-GIC	Glass ionomer cement containing expanded-pore
PolyB	Polymyxin B
RB	Rose bengal
ROS	Reactive oxygen species
*S. aureus*	*Staphylococcus aureus*
*S. enterica*	*Salmonella enterica*
*S. epidermidis*	*Staphylococcus epidermidis*
*S. mutans*	*Streptococcus mutans*
*S. oralis*	*Streptococcus oralis*
SB	Schiff base
SBA-15 Ag/MSNPs	Mesoporous silica nanoparticles with hexagonal mesoporous structure and microporous connections decorated with silver NPs
SBA-15 DAMO-MSM	SBA15 mesoporous silica material functionalized with (*N*-(2-aminoethyl)-3-aminopropyltrimethoxysilane)
SBA-15 PMMA/MSNPs	Poly(methyl methacrylate) resine containing SBA-15 MSNPs
SBA-15	Mesoporous structure with a hexagonal pattern and microporous connections
SEM	Scanning electron microscopy
Si-Ti-Sv	Silica-titania sieves
TC	Tetracycline
TEM	Transmission electron microscopy
Tre	Trehalose
Tre-HOMSNPs	Trehalose functionalized Hollow oblate mesoporous silica nanoparticles
UL-MSNPs	MSNPs coated with a liposome bilayer and functionalized with the ubiquicidin peptide (UBI_29–41_)
UV	Ultra violet
Van	Vancomicin
ε-pLys	ε-poly-l-lysine cationic polymer
